# RNase H1 Regulates Mitochondrial Transcription and Translation *via* the Degradation of 7S RNA

**DOI:** 10.3389/fgene.2019.01393

**Published:** 2020-01-31

**Authors:** Aurelio Reyes, Joanna Rusecka, Katarzyna Tońska, Massimo Zeviani

**Affiliations:** ^1^ MRC Mitochondrial Biology Unit, University of Cambridge, Cambridge, United Kingdom; ^2^ Institute of Genetics and Biotechnology, Faculty of Biology, University of Warsaw, Warsaw, Poland

**Keywords:** mitochondria, mtDNA, mitochondrial disease, RNase H1, transcription, translation, 7S DNA, 7S RNA

## Abstract

RNase H1 is able to recognize DNA/RNA heteroduplexes and to degrade their RNA component. As a consequence, it has been implicated in different aspects of mtDNA replication such as primer formation, primer removal, and replication termination, and significant differences have been reported between control and mutant *RNASEH1* skin fibroblasts from patients. However, neither mtDNA depletion nor the presence of deletions have been described in skin fibroblasts while still presenting signs of mitochondrial dysfunction (lower mitochondrial membrane potential, reduced oxygen consumption, slow growth in galactose). Here, we show that RNase H1 has an effect on mtDNA transcripts, most likely through the regulation of 7S RNA and other R-loops. The observed effect on both mitochondrial mRNAs and 16S rRNA results in decreased mitochondrial translation and subsequently mitochondrial dysfunction in cells carrying mutations in *RNASEH1*.

## Introduction

Human mitochondrial DNA (mtDNA) encodes 2 rRNAs, 22 tRNAs, and 13 out of 83 proteins that are subunits of the respiratory chain, while the remaining proteins required for mitochondrial function are encoded in the nucleus. Indeed, all proteins responsible for mtDNA maintenance, especially those involved in replication, as well as other proteins necessary for transcription and translation, are encoded in the nucleus (Gustafsson et al., 2016). Human mtDNA replication requires several factors that constitute the replisome and that include DNA polymerase subunits POLG and POLG2, the helicase TWNK, the single-stranded binding protein SSBP1, and DNA topoisomerases TOP1, TOP2A and TOP2B (Gustafsson et al., 2016). The nucleases MGME1, DNA2, FEN1, and RNase H1 have been described in mitochondria, and they have been related to mtDNA replication, especially but not exclusively with regard to primer removal (Kazak et al., 2013; Uhler and Falkenberg, 2015; Al-Behadili et al., 2018; Posse et al., 2019).

The nuclease RNase H1 can be targeted to both the nucleus and mitochondria, and it is able to recognize DNA/RNA heteroduplexes and to degrade their RNA component (Suzuki et al., 2010). The enzyme consists of three domains: a hybrid binding domain and a catalytic domain separated by a connecting domain (Nowotny et al., 2007). The hybrid binding domain is responsible for the recognition of the DNA/RNA hybrids, and it also enhances both the specific activity and the processivity of the enzyme (Nowotny et al., 2008). Despite not being essential, the presence of this domain results in a protein with greater binding affinity and positional preference for cleavage than the bacterial counterpart (Wu et al., 2001). The catalytic domain is very conserved from bacteria to humans and it contains key residues of the activity (Nowotny et al., 2007). The connecting domain has been less characterized, but it has been described to be required for RNase H activity (Wu et al., 2001).

In the nucleus, RNase H1 activity has been linked to the removal of R-loops (nascent RNA hybridized to template DNA with a single-stranded non-template DNA) in rDNA (Shen et al., 2017) and immunoglobulin sites (Parajuli et al., 2017), Okazaki fragment processing (Lima et al., 2007), DNA repair (Tannous et al., 2015; Amon and Koshland, 2016), telomere elongation (Arora et al., 2014), and hypermutability in the immunoglobulin locus (Maul et al., 2017). In mitochondria, RNase H1 has been implicated in different aspects of mtDNA replication such as replication initiation at origin-specific sites (Posse et al., 2019) and primer removal at both origins of replication (Holmes et al., 2015; Reyes et al., 2015; Al-Behadili et al., 2018), segregation of daughter mtDNA molecules post replication (Akman et al., 2016), R-loop processing (Reyes et al., 2015; Lima et al., 2016; Gonzalez de Cozar et al., 2019), and processing of mitochondrial ribosomal RNA precursor (Wu et al., 2013).

Mutations in genes involved in mitochondrial genome stability result in mtDNA depletion, large-scale multiple deletions, or accumulation of point mutations, which, in turn, can lead to mitochondrial diseases (Almannai et al., 2018; Rusecka et al., 2018). In the past few years, 15 patients with mitochondrial diseases have been found to carry mutations in the *RNASEH1* gene, mainly as compound heterozygous c.424G > A (p.Val142Ile) and c.469C > T (p.Arg157*) (Reyes et al., 2015), c.424G > A (p.Val142Ile) and c.554C > T (p.Ala185Val) (Reyes et al., 2015), c.424G > A (p.Val142Ile) and c.442T > C (p.Cys148Arg) (Bugiardini et al., 2017; Sachdev et al., 2018), and c.487T > C (p.Tyr163His) and c.258_260del (p.Gln86del) (Carreno-Gago et al., 2019) but in some cases as homozygous c.424G > A (p.Val142Ile) (Reyes et al., 2015; Akman et al., 2016). All mutations mapped in the catalytic domain, except c.258_260del (p.Gln86del), which mapped in the connecting domain. Affected individuals presented with adult-onset chronic progressive external ophthalmoplegia (CPEO), ptosis, dysphagia, muscle weakness, ataxia, and respiratory impairment. Mitochondrial DNA depletion and multiple deletions, COX-deficient fibers and low complex I and IV activities are characteristic features of the muscle biopsies from the patients with *RNASEH1* mutations (Reyes et al., 2015; Bugiardini et al., 2017; Sachdev et al., 2018; Carreno-Gago et al., 2019). However, neither significant mtDNA depletion nor the presence of multiple deletions have been observed in skin fibroblasts derived from these patients (Reyes et al., 2015; Akman et al., 2016; Carreno-Gago et al., 2019). Despite this, *RNASEH1* mutant fibroblasts presented lower mitochondrial membrane potential, reduced oxygen consumption, and slower growth than control fibroblasts (Reyes et al., 2015; Reyes et al., 2018). Therefore, RNase H1 may have additional roles not related to mtDNA maintenance that could be held responsible for this phenotype.

In this paper, we show that RNase H1 plays an important role in mtDNA transcription. Mutant *RNASEH1* skin fibroblasts showed a significant decrease in some mitochondrial transcripts, e.g., MT-CO2, MT-ND5, and MT-RNR2 (16S rRNA). Interestingly, the levels of 7S RNA (MT-7S), a small non-coding mitochondrial transcript, were also upregulated in the patient fibroblasts. 7S RNA is involved in the primer synthesis required for mtDNA replication but it has also been suggested to play a role as a negative regulator of mtDNA transcription (Cantatore et al., 1988). Hence, the decrease of transcript levels in the patient fibroblasts could be related to the increase in 7S RNA, as this may not have been efficiently removed by the lower levels and activity of mutant RNase H1 in the patient. In addition, a lack of or slow processing of R-loops in different regions of mtDNA could also affect transcript levels. A decrease in mitochondrial translation due to a decrease in 16S rRNA and possible direct interaction of 7S RNA with 12S rRNA could also explain the mitochondrial dysfunction we detected in these cells.

## Materials and Methods

### Structural Modeling of Mutant RNase H1

The crystal structure of the human RNase H1 catalytic domain in a complex with 18-mer DNA/RNA heteroduplex (PDB ID 2QK9) was downloaded from the Protein Data Bank (PDB) database and loaded onto PyMOL. Conserved residues previously reported to constitute the active site of the protein (Nowotny et al., 2007) were manually colored in yellow and visualized as sticks, while the DNA and RNA components of the heteroduplex were colored in cyan and magenta, respectively. The mutagenesis option available in PyMOL was used to replace Val^142^ with Ile^142^. These two residues and the neighboring residue Trp^164^ were displayed in different colors and visualized as sticks in order to highlight the possible effect the mutation could have on the structure of the protein.

### Cell Culture Conditions

Fibroblasts derived from skin biopsy were obtained from a patient (P) carrying two pathogenic mutations in the *RNASEH1* gene (GenBank: NM_002936.4): c.424G > A (p.Val142Ile) on the paternal allele and a nonsense mutation, c.469C > T (p.Arg157*), on the maternal allele (Reyes et al., 2015). In addition, control fibroblasts were obtained from two healthy controls (C1 and C2). Fibroblast cell lines were maintained in high-glucose medium (Gibco) supplemented with 10% FBS (Gibco) and 1% penicillin-streptomycin at 37°C in a humidified atmosphere of 5% CO_2_. Primary skin fibroblasts were immortalized by lentiviral transduction of pLOX-Ttag-iresTK (Addgene #12246, Tronolab), as previously described (Reyes et al., 2018). Briefly, human 293T cells were cotransfected with transfer vector (pLOX-Ttag-iresTK), second-generation packaging plasmid (pCMVdR8.74), and envelope plasmid (pMD2.VSVG) (Naldini et al., 1996). Infectious lentiviral particles were collected from the medium 24 h after transfection and used for transduction of all three fibroblast cell lines. Transduced fibroblasts were grown for at least six passages in order to make sure immortalized cells were selected. Changes in cell shape and doubling time were observed as part of the normal process of immortalization. All experiments here reported were carried out on immortalized fibroblasts. When required, high-glucose medium was replaced by glucose-free medium (Gibco) and supplemented with 50 mM galactose (Sigma).

### Immunoblot Analysis

Protein gel electrophoresis and blotting analyses were performed on whole cell protein extracts obtained from patient (P) and control (C1 and C2) fibroblasts. Samples containing 30 μg protein were separated by denaturing NuPAGE 4%–12% Bis-Tris gels and transferred to nitrocellulose membrane. Immunodetection was carried out using primary antibodies against target proteins: RNase H1 (ab56560, Abcam), POLG (sc-5931, Santa Cruz), POLG2 (LS-C334882, LSBio), TWNK (gift from M Falkenberg), SSBP1 (ab74710, Abcam), TFAM (gift from RJ Wiesner), POLRMT (ab32954, Abcam), LRPPRC (ab97505, Abcam), SLIRP (ab51523, Abcam), ATAD3 (gift from JE Walker), bL12 (14795-1-AP, Proteintech), uL11 (SAB2701374, Sigma), MDDX28 (ab70821, Abcam), mS35 (16457-1-AP, Proteintech), mS18b (16139-1-AP, Proteintech), NDUFS3 (ab110246, Abcam), NDUFB8 (ab110242, Abcam), SDHA (ab14715, Abcam), SDHB (ab14714, Abcam), UQCRC1 (ab96333, Abcam), UQCRC2 (ab14745, Abcam), MT-CO1 (ab14705, Abcam), MT-CO2 (ab91317, Abcam), COX4l1 (ab14744, Abcam), ATPF1 (ab84625, Abcam), and ATPA1 (ab110273, Abcam), along with GAPDH (ab8245, Abcam), used as loading control. For quantifications, images were digitalized and analyzed with ImageJ software, and data analyses were performed in Microsoft Excel.

### DNA Isolation, Gel Electrophoresis, and Hybridization

Total DNA from patient (P) and control (C1 and C2) fibroblasts was extracted using Wizard Genomic Purification Kit (Promega). Total DNA (5 μg) was digested with *Pvu*II (NEB), and the fragments were resolved in 1% agarose gels. After electrophoresis and Southern blot, hybridizations with radiolabelled probes directed against the human mtDNA (nucleotide positions 16,341-151) and nuclear 18S rDNA were carried out overnight at 65°C in 7% SDS and 0.25M sodium phosphate buffer pH 7.4. After washing four times with 1x SSC (150 mM sodium chloride, 15 mM sodium citrate, pH 7.0) and twice with 1 x SSC/0.1% SDS, membranes were exposed to Phosphorimager screens for 0.5 to 10 days. ImageQuant software was used for the quantification of the signal.

### RNA Isolation and Quantitative PCR (qPCR)

Total RNA from patient (P) and control (C1 and C2) fibroblasts was extracted using Trizol (Invitrogen). RNA was then treated with DNase I (DNA-free kit, Ambion) and reverse transcribed with Omniscript reverse transcription kit (Qiagen). Quantitative polymerase chain reaction (qPCR) analyses were performed with Life Technologies Gene Expression Assays (Applied Biosystems): RNase H1 (Hs00268000_m1, Hs01108220_g1 and Hs01108219_g1 on exons 7-8, 2-3 and 1-2 boundary, respectively), MT-7S (7S RNA, Hs02596861_s1), MT-RNR1 (12S rRNA, Hs02596859_g1), MT-RNR2 (16S rRNA, Hs02596860_s1), MT-CO1 (Hs02596864_g1), MT-CO2 (Hs02596865_g1), MT-CO3 (Hs0259866_g1), MT-ND1 (Hs02596873_s1), MT-ND5 (Hs02596878_g1), MT-ND6 (Hs02596879_g1), MT-CYB (Hs02596867_s1), and MT-ATP6 (Hs02596862_g1) and normalized to levels of GAPDH (Hs02758991_g1).

### Mitochondrial Translation

Patient (P) and control (C1 and C2) fibroblast cell lines were subjected to metabolic labeling of mtDNA encoded proteins. [^35^S]-methionine was added to the medium after treatment with emetine dihydrochloride and labeling was performed for 1 h, as previously described (Chomyn, 1996). Cells were lysed and proteins (30 µg) were loaded onto 12% polyacrylamide gels. Gels were stained with Coomassie blue, dried, and then exposed to Typhoon phosphor screens, with products visualized and quantified with ImageQuant software (GE Healthcare).

### Oxygen Consumption

Respiration in patient (P) and control (C1 and C2) fibroblasts, *I_O2_* [pmols·s^-1^·10^-6^ cells], was calculated as the negative time derivate of oxygen concentration as measured by the OROBOROS Oxygraph-2k on one million cell/ml in a 2-ml chamber at 37°C. Basal respiration was measured without substrates, and the proton leak state after the addition of oligomycin (50nM) was also measured. Oxygen consumption coupled to ATP production was calculated as the difference between basal respiration and proton leak. Maximal respiration was measured by stepwise 1.25 µM titration of CCCP and inhibition by 2 µM rotenone and 2.5 µM antimycin A for the final measurement of residual oxygen consumption. Spare capacity was calculated as the difference between maximal respiration and basal respiration.

### Mitochondrial Membrane Potential

Mitochondrial membrane potential was measured in patient (P) and control (C1 and C2) untreated fibroblasts and after treatment with 1 µM FCCP for 5 min at 37°C as the ratio of the red to the green JC-1 signal using a Nucleo Counter NC-3000 Advanced Image Cytometer.

### Statistics

Fibroblasts from a single patient with mutations in *RNASEH1* and two non-related healthy individuals were analyzed as controls. All numerical data are expressed as mean ± standard deviation of the mean (SD). Student’s unpaired two-tailed t-tests under the assumption of a normal distribution and unequal variance were used for statistical analysis combining the data from both controls against the patient unless specified otherwise. Control 1 (C1) fibroblasts were randomly chosen as the reference for all experiments, the values obtained in the first biological repeat were arbitrarily assigned as 1 and, subsequently, all other values were corrected accordingly.

## Results

### Characterization of the Mutations in *RNASEH1*

**Figure 1:**
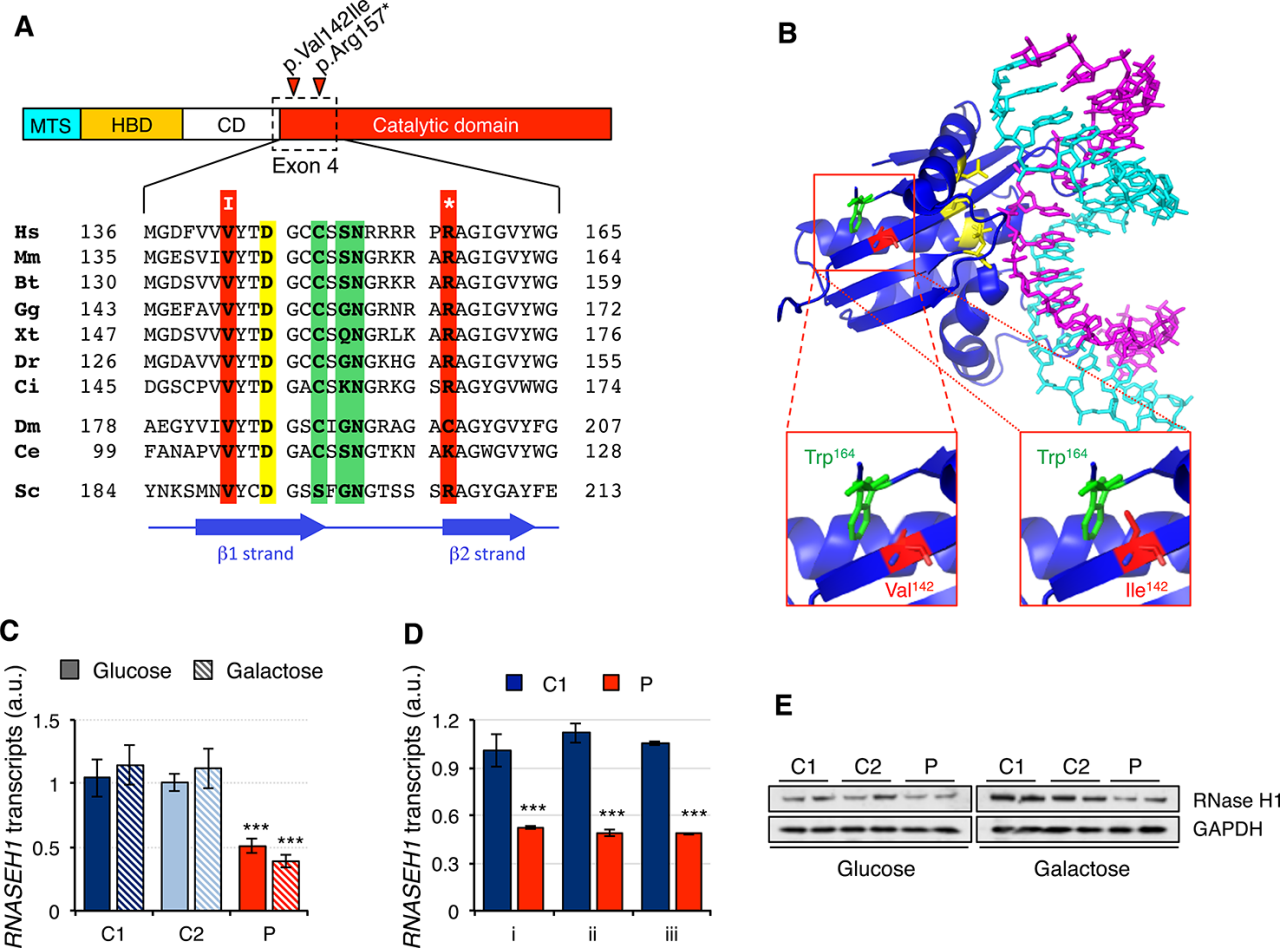
*RNASEH1* mutations, transcript, and protein levels. **(A)** Domains ofhuman RNAse H1 protein (MTS, mitochondrial targeting sequence; HBD, hybrid binding domain; CD, connection domain; catalytic domain). RNase H1 protein sequences from representative species,*H. sapiens* (Hs, NP_002927) *M. musculus* (Mm, NP_035405), *B. taurus* (Bt, NP_001039970), *G. gallus* (Gg, NP_990329),*X. tropicalis* (Xt, NP_001096299),*D. rerio* (Dr, NP_001002659), *C. intestinalis* (Ci, F6QPH0), *D. melanogaster* (Dm, NP_995777), *C. elegans* (Ce, NP_001040786), *S. cerevisiae* (Sc, Q04740), were extracted from the database and aligned using ClustalW2. Conserved residues found mutated in the patient in exon 4 are boxed in red, while residues in the active site and interacting with DNA or RNA are boxed in yellow and green, respectively. Positions of β strands are marked by blue arrows. **(B)** Human RNase H1 crystal structure (PDB ID 2QK9) 18 bp DNA(cyan): RNA (magenta) hybrid is shown respectively. Residues in the active site are colored in yellow. Residues Trp^164^ (green) and Val^142^, or the mutated variant Ile^142^ (red), are shown as sticks. **(C)** *RNASEH1* transcript levels in control (C1 and C2) and patient (P) fibroblasts grown in either glucose- or galactose-containing medium assessed by qPCR (probe Hs00268000_m1) and normalized to *GAPDH* transcript levels. Data are shown as mean ± SD, n = 4, ***p < 0.001. **(D)** *RNASEH1* transcript levels in control (C1) and patient (P) fibroblasts grown in glucose-containing medium assessed by qPCR with probes Hs00268000_m1 (i), Hs01108220_g1 (ii), and Hs01108219_g1 (iii) and normalized to *GAPDH* transcript levels. Data are shown as mean ± SD, n = 3, ***p < 0.001. **(E)** Western blot analysis of RNase H1 in control (C1 and C2) and patient (P) fibroblasts grown in either glucose- or galactose-containing medium. GAPDH was used as loading control.

The two mutations present in the *RNASEH1* gene in the patient were first analyzed *in silico*. The missense mutation, c.424G > A (p.Val142Ile), involved a residue in a conserved position of the β1 strand, close to one of the four key catalytic residues (**Figure 1A**). Modeling of the mutation on the crystal structure of human RNase H1 (PDB ID 2QK9) showed that Ile^142^ is a bulkier residue than Val^142^ and therefore could interfere with another bulky residue nearby, Trp^164^, causing a change in the orientation of the β1 strand (**Figure 1B**). This could result in a misalignment of the four catalytic residues that constitute the active site and/or the residues involved in the interaction with the DNA/RNA hybrids. The nonsense mutation, c.469C > T (p.Arg157*), affects a residue at the N-terminus of the catalytic domain and, as a consequence, the truncated protein is void of any activity (Reyes et al., 2015). Nonsense-mediated decay is a conserved quality control mechanism that selectively degrades the transcripts harboring premature stop codons (Kurosaki et al., 2019). In order to investigate if the presence of a nonsense mutation was triggering nonsense-mediated decay, we checked *RNASEH1* transcript levels in human control (C1 and C2) and patient (P) fibroblasts grown in either glucose- or galactose-containing medium with probe Hs00268000_m1, spanning exons 7-8 (**Figure 1C**). *RNASEH1* transcript levels were significantly reduced to at least 50% of controls in both growing conditions. The same results were obtained when different probes upstream of the nonsense mutation were used, Hs01108220_g1 and Hs01108219_g1, spanning exons 2-3 and 1-2, respectively (**Figure 1D**), further supporting nonsense-mediated decay. As a consequence of the decrease in transcript levels, a significant decrease was also observed at protein levels in patient fibroblasts (**Figure 1E**).

### Mitochondrial DNA-Related Alterations in Patient Fibroblasts

**Figure 2:**
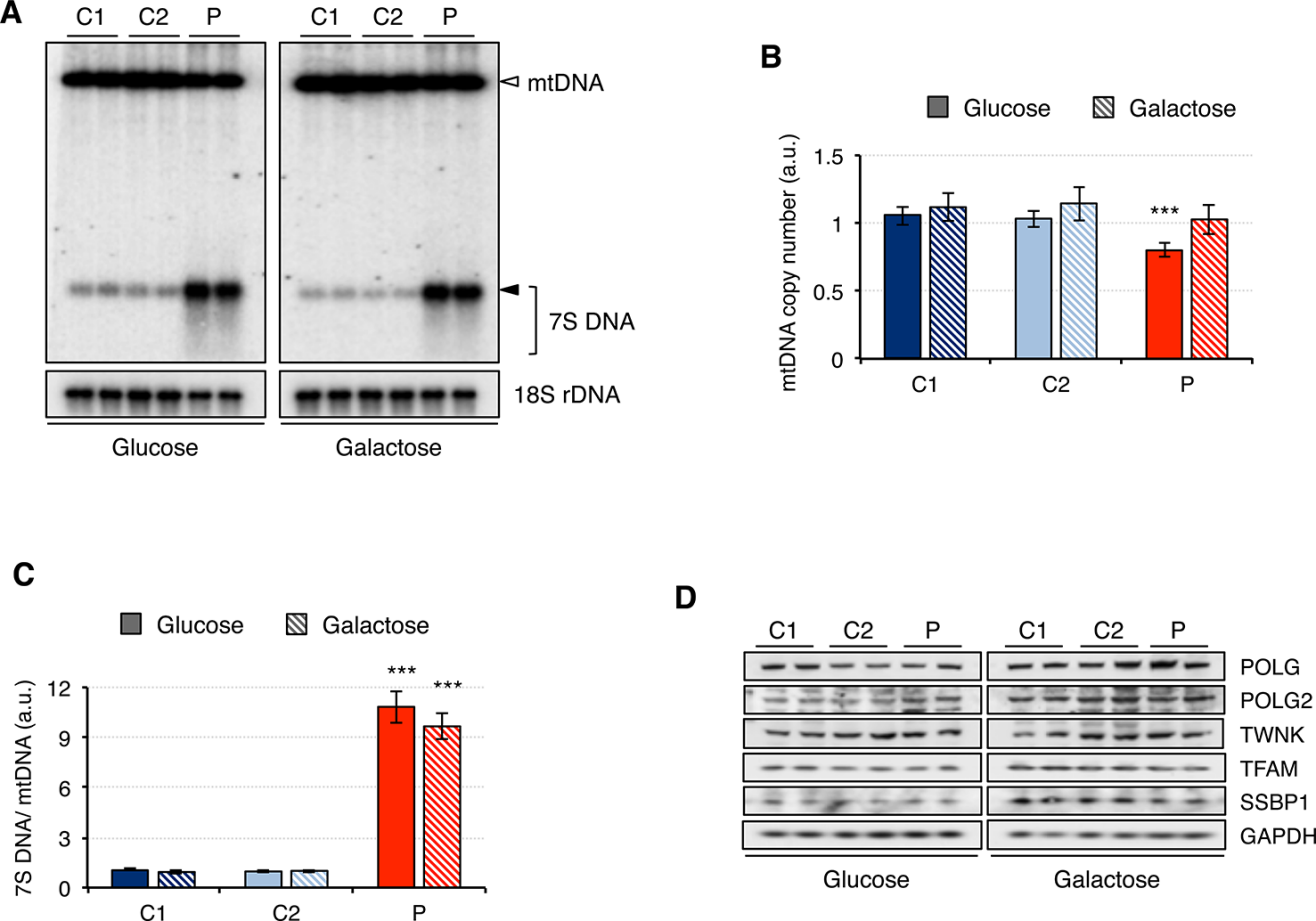
Mitochondrial DNA maintenance in mutant *RNASEH1* fibroblasts. **(A)** Southern blot of total DNA digested with *Pvu*II from control (C1 and C2) and patient (P) fibroblasts grown in either glucose- or galactose-containing medium. A radioactive probe against mtDNA was used to detect both linearized mtDNA (empty arrowhead) and 7S DNA (filled arrowhead and bracket), while a probe against 18S rDNA was used as loading control. **(B)** Relative mitochondrial DNA copy number in control (C1 and C2) and patient (P) fibroblasts grown in either glucose- or galactose-containing medium, calculated as the linearized mtDNA/18S rDNA signal ratio. Data are shown as mean ± SD, n = 4, ***p < 0.001. **(C)** 7S DNA levels in control (C1 and C2) and patient (P) fibroblasts grown in either glucose- or galactose-containing medium calculated as the 7S DNA/linearized mtDNA/18S rDNA signal ratio. Data are shown as mean ± SD, n = 4, Student’s unpaired two-tail t-test, ***p < 0.001. **(D)** Western blot analysis of mitochondrial proteins involved in mtDNA maintenance in control (C1 and C2) and patient (P) fibroblasts grown in either glucose- or galactose-containing medium. GAPDH was used as loading control.

Since analysis of muscle biopsy from patients carrying mutations in *RNASEH1* has revealed the presence of multiple deletions and depletion in mtDNA (Reyes et al., 2015; Bugiardini et al., 2017; Carreno-Gago et al., 2019), we performed a Southern blot on genomic DNA extracted from control (C1 and C2) and patient (P) fibroblasts grown in either glucose or galactose (**Figure 2A**). No deletions on mtDNA were detected in the patient fibroblasts, and the mtDNA copy number was only marginally reduced to 80% compared to controls when the cells were grown in glucose, with no significant difference observed when cells grew in galactose (**Figures 2A, B**). By contrast, 7S DNA, the third strand of the mtDNA displacement loop, was 10-fold higher in the patient fibroblasts than in control, both in glucose and galactose (**Figures 2A, C**). Furthermore, 7S DNA in controls appears as a net band, as all the molecules have the same length, while in the patient fibroblasts, there is a smear below the main band, indicating that some 7S DNA molecules are shorter than the expected size (**Figure 2A**). This effect is more pronounced in glucose than in galactose.

Then we analyzed the steady-state level of mitochondrial proteins involved in mtDNA maintenance (**Figure 2D**). Other than the overall increase in the steady-state level of all proteins when cells were grown in galactose medium, no significant differences between patient and control fibroblasts were observed. These results are in agreement with the observed minor changes in mtDNA copy number in the patient fibroblasts (**Figure 2A**).

### Mitochondrial RNA-Related Alterations in Patient Fibroblasts

**Figure 3:**
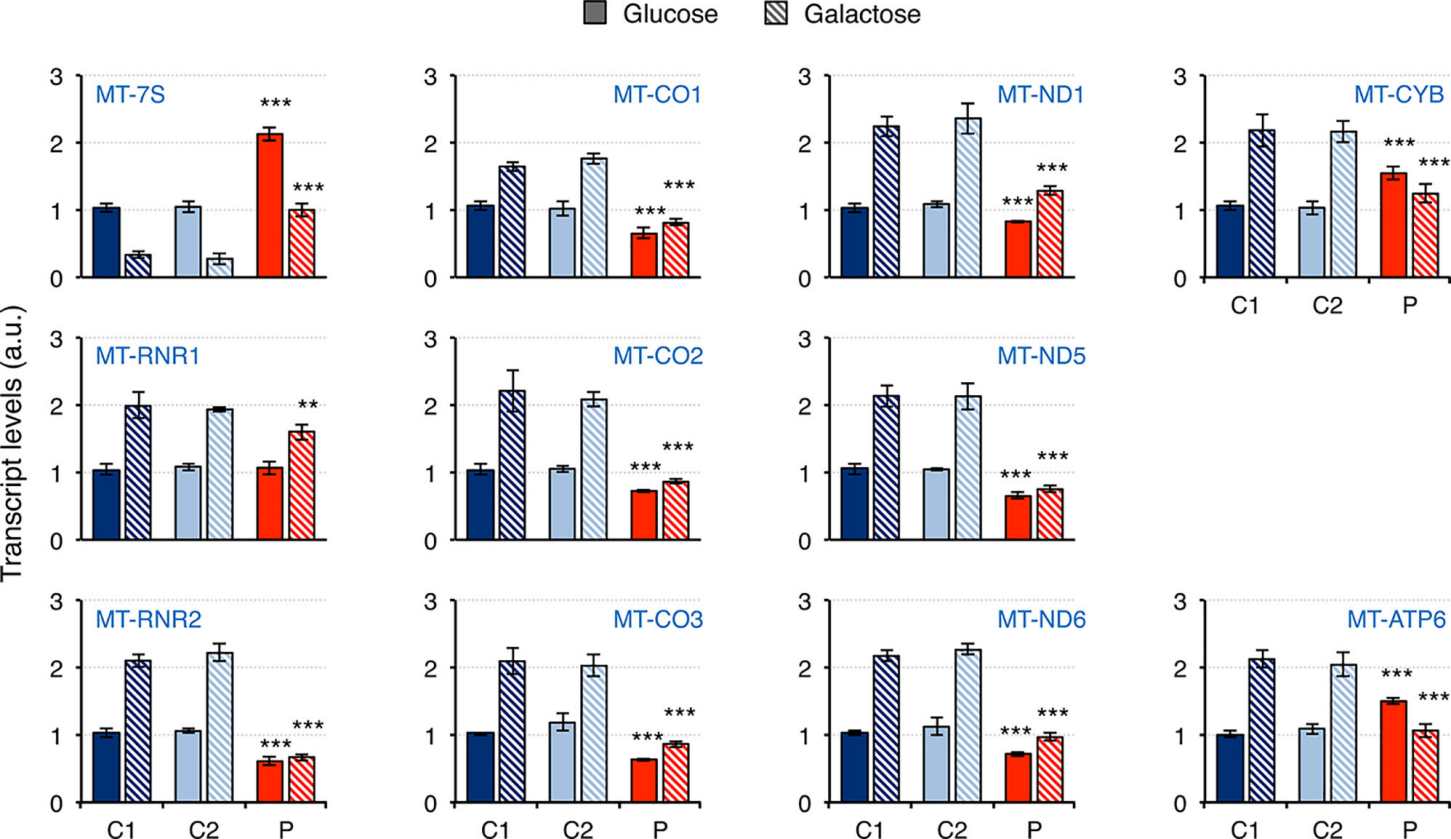
Mitochondrial transcript levels in mutant *RNASEH1* fibroblasts. Mitochondrial transcript levels in control (C1 and C2) and patient (P) fibroblasts grown in either glucose- or galactose-containing medium, assessed by qPCR and normalized to *GAPDH* transcript levels. Analyzed transcripts included the non-coding 7S RNA (MT-7S), the two ribosomal RNAs MT-RNR1 (12S rRNA) and MT-RNR2 (16S rRNA), three complex IV protein mRNAs (MT-CO1, MT-CO2 and MT-CO3), three complex I protein mRNAs (MT-ND1, MT-ND5 and MT-ND6), one complex III protein mRNA (MT-CYB), and one complex V protein mRNA (MT-ATP6). Data are shown as mean ± SD, n = 4, Student’s unpaired two-tail t-test, **p < 0.01, ***p < 0.001.

Mitochondrial dysfunction has been reported in fibroblasts from patients carrying mutations in *RNASEH1*, but neither mtDNA deletions nor depletion have been observed (Reyes et al., 2015; Bugiardini et al., 2017; Carreno-Gago et al., 2019). Therefore, we first investigated whether there was an effect on mitochondrial transcription. The steady-state levels of 11 transcripts was analyzed by qPCR and included 7S RNA (MT-7S), the two ribosomal RNAs MT-RNR1 (12S rRNA) and MT-RNR2 (16S rRNA), and eight protein mRNAs from all four different oxidative phosphorylation (OxPhos) complexes with mitochondrially-encoded subunits: CI, CIII, CIV, and CV (**Figure 3**). The non-coding 7S RNA is a polyadenylated transcript of about 200 nt whose 5’ end maps at the light strand promoter (LSP) and has been implicated in both mtDNA replication and transcription. Transcript levels of 7S RNA in galactose medium were lower than in glucose in all cell lines. Moreover, a two-fold and three-fold increase in 7S RNA was detected in patient fibroblasts compared to controls grown in glucose and galactose, respectively. As a result, patient cells grown in galactose had the same levels of 7S RNA as controls grown in glucose. For all the other transcripts analyzed, the levels in control cells growing in galactose medium were always higher than in glucose medium, suggesting increased mitochondrial biogenesis. In the case of patient fibroblasts, the results varied depending on the transcript. The two ribosomal rRNAs showed a different behavior: a slight decrease in 12S rRNA was observed in patient cells only when they were grown in galactose medium, while a significant decrease in 16S rRNA compared to controls was observed both in glucose and galactose growth (40% and 80% decrease, respectively). The transcripts of both complex IV (MT-CO1, MT-CO2, MT-CO3) and complex I (MT-ND1, MT-ND5, MT-ND6) subunits were moderately decreased in patient cells grown in glucose medium (28-36% and 17-35% decrease for complex IV and I, respectively), and culture in galactose medium did not increase their levels very significantly in most of the cases, which increased the difference with control cell lines (40-43% and 35-50% decrease for complex IV and I, respectively). A completely different trend was observed in transcripts from complex III (MT-CYB) and complex V (MT-ATP6) mitochondrial subunits: transcript levels in glucose growth were higher in the patient fibroblasts than in controls (about 50% increase in both cases), while in galactose growth, they were lower compared to controls (about 50% decrease in both cases).

**Figure 4:**
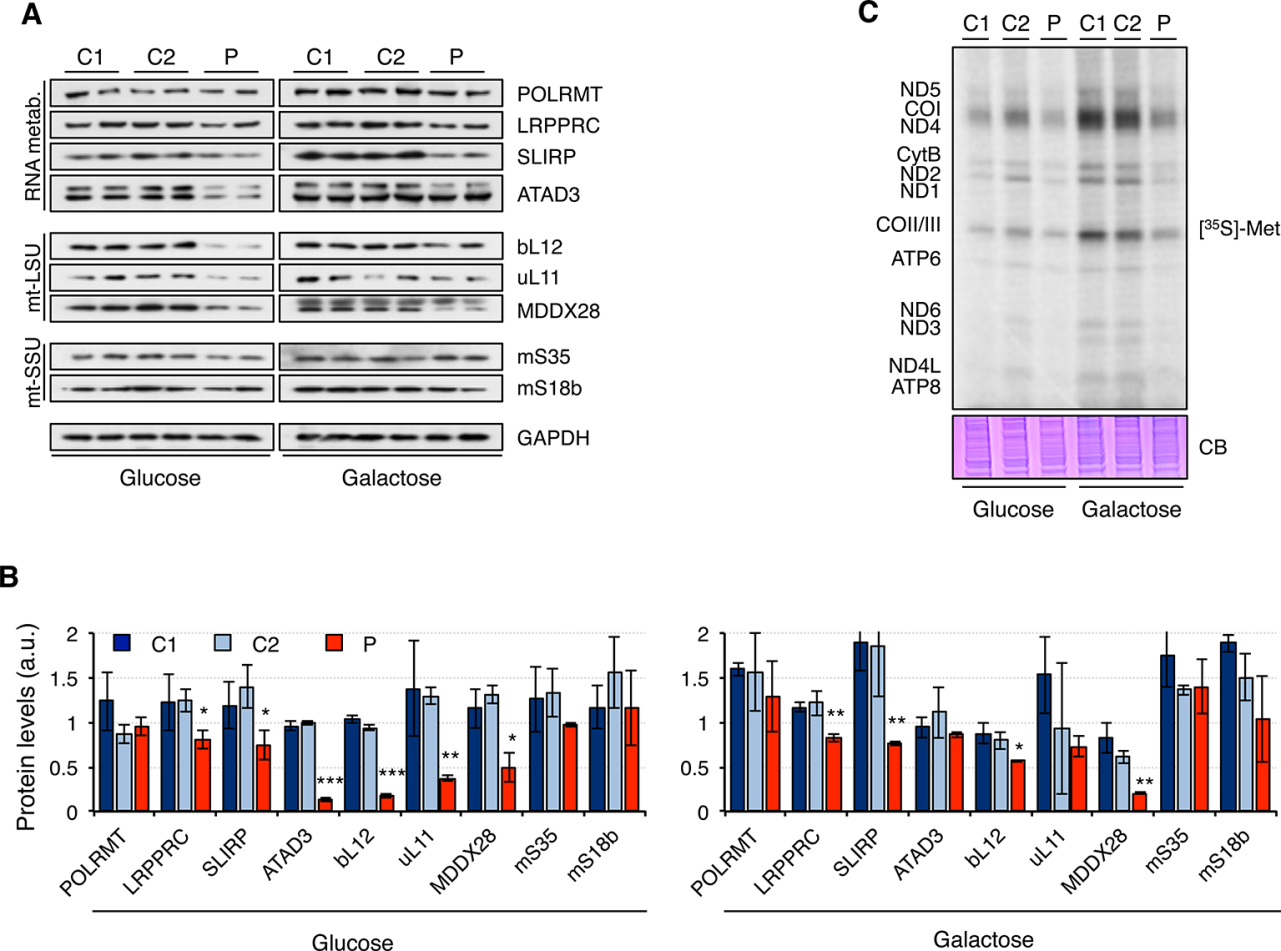
Mitochondrial translation in mutant *RNASEH1* fibroblasts. **(A)** Western blot analysis of mitochondrial proteins involved in mitochondrial RNA metabolism (RNA metab.) and mitochondrial large (mtLSU) and small (mtSSU) ribosomal subunits in control (C1 and C2) and patient (P) fibroblasts grown in either glucose- or galactose-containing medium. GAPDH was used as loading control. GAPDH is from the same blot as **Figure1D**. **(B)** Quantification of the Western blots shown in **(A)** normalized to GAPDH levels. Data are shown as mean ± SD, Student’s unpaired two-tail t-test, *p < 0.05, **p < 0.01, ***p < 0.001. **(C)** [^35^S]-methionine *de novo* synthesis of mitochondrially encoded proteins in control (C1 and C2) and patient (P) fibroblasts grown in either glucose- or galactose-containing medium. Newly synthesized proteins were visualized after exposure of the dried gel to phosphor screens. The coomassie blue (CB) staining shown below was used as loading control. n = 3.

Next, we analyzed the steady-state levels of proteins involved in RNA metabolism and mitochondrial ribosomal proteins (**Figure 4A**). Overall, protein levels were higher in galactose than in glucose medium, suggesting increased mitochondrial biogenesis. While the mitochondrial RNA polymerase, POLRMT, was not significantly changed in patient fibroblasts, other proteins such as LRPPRC, SLIRP, and ATAD3 were decreased in patient fibroblasts, particularly when grown in glucose medium. Moreover, mitochondrial ribosomal proteins from the large 39S subunit (mt-LSU) but not the small 28S subunit (mt-SSU) were also found to be decreased in patient fibroblasts growing in glucose medium and, to a lesser extent, also in galactose medium (**Figures 4A, B**). As a consequence, mitochondrial translation was impaired in patient fibroblasts, with all mitochondrial proteins equally affected (**Figure 4C**).

### Mitochondrial Dysfunction in Patient Fibroblasts

**Figure 5:**
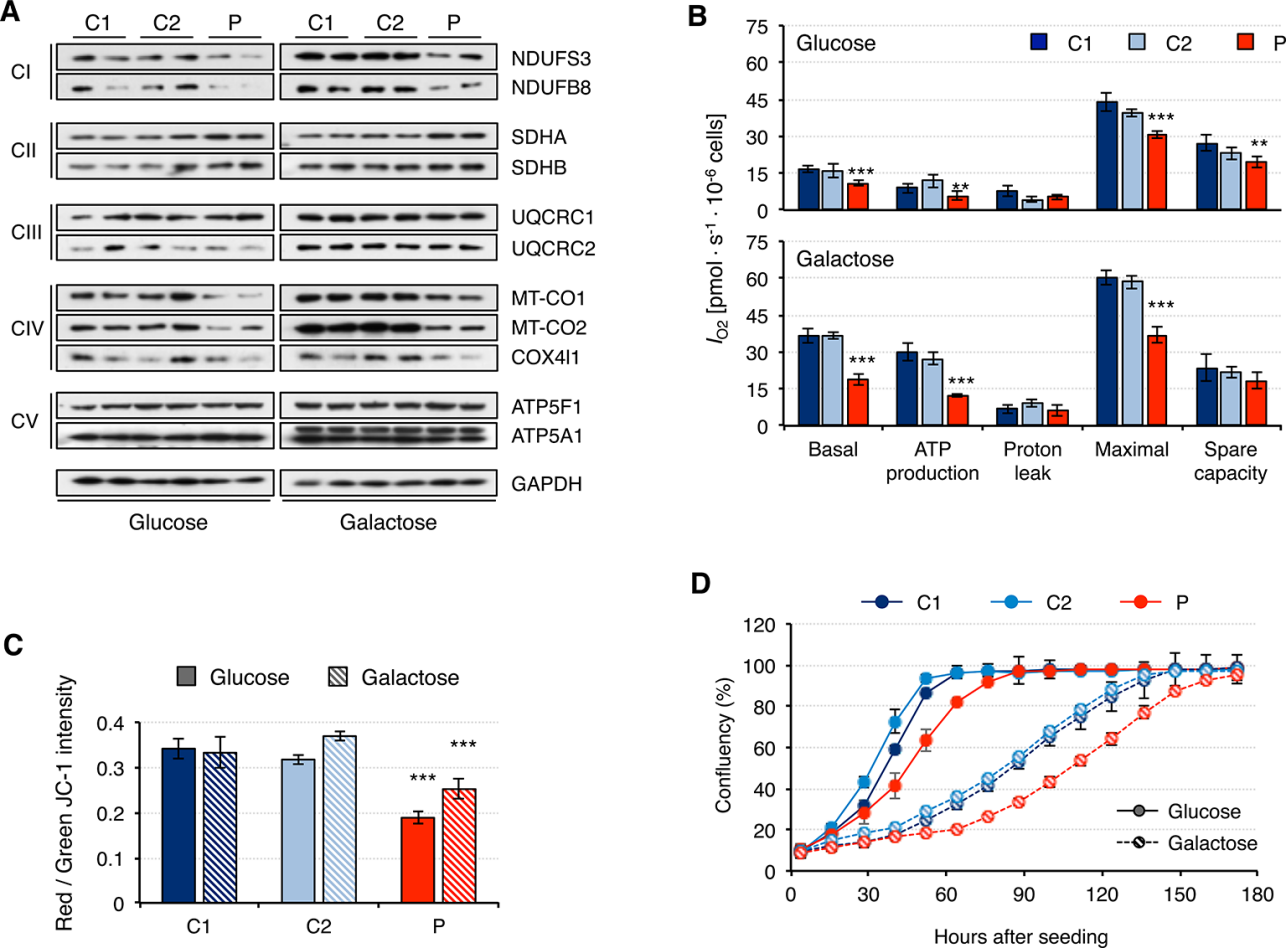
Mitochondrial DNA maintenance in mutant *RNASEH1* fibroblasts. **(A)** Western blot analysis of representative components of the mitochondrial OxPhos complexes I-V in control (C1 and C2) and patient (P) fibroblasts grown in either glucose- or galactose-containing medium. GAPDH was used as loading control. **(B)** Oxygen consumption (*I*_O2_) measurements in control (C1 and C2) and patient (P) fibroblasts grown in either glucose- or galactose-containing medium. Values of basal and maximal respiration along with ATP production-dependent, proton leak respiration, and spare capacity are presented. Data are shown as mean ± SD, n = 4, Student’s unpaired two-tail t-test, **p < 0.01, ***p < 0.001. **(C)** Mitochondrial membrane potential in control (C1 and C2) and patient (P) fibroblasts grown in either glucose- or galactose-containing medium using JC-1 staining. Data are shown as mean ± SD, n = 4, Student’s unpaired two-tail t-test, ***p < 0.001. **(D)** Growth curves of control (C1 and C2) and patient (P) fibroblasts grown in either glucose or galactose. Cell growth was monitored continuously in an Incucyte cell imager (Essen Bioscience). Data correspond to one of the three independent experiments carried out, and they are shown as the mean of three technical replicates ± SD.

Fibroblasts from patients carrying mutations in *RNASEH1* have signals of mitochondrial dysfunction (Reyes et al., 2015; Bugiardini et al., 2017; Carreno-Gago et al., 2019). Indeed, we have shown that patient fibroblasts have alterations in mitochondrial transcription and translation that could lead to mitochondrial dysfunction. Therefore, we first analyzed the steady-state of the OxPhos constituents of all five complexes (**Figure 5A**). Again, overall protein levels were higher in galactose than in glucose medium, supporting increased mitochondrial biogenesis. Patient fibroblasts presented lower steady-state levels of all analyzed subunits of complex I and complex IV, while no difference was detected for complexes III and V. These results are in agreement with the data from mitochondrial transcript levels (**Figure 3**). Complex II subunits were slightly increased in patient fibroblasts compared to controls, most likely as a compensation mechanism. A consequence of the observed decrease in OxPhos protein levels was an alteration in mitochondrial respiration, as measured by oxygen consumption, *I*_O2_. Patient fibroblasts showed significant lower basal *I*_O2_, ATP-dependent, and maximal *I*_O2_, both in glucose and galactose (**Figure 5B**). Mitochondrial membrane potential is usually altered in cases of a dysfunctional electron transport chain and, indeed, we observed a significant decrease in membrane potential in patient fibroblasts both in glucose and galactose (**Figure 5C**). These mitochondrial alterations in the patient fibroblasts have many consequences at a cellular level, and a lower growth rate is one of them (**Figure 5D**).

## Discussion

Pathological mutations in *RNASEH1* have been described in patients with mitochondrial depletion and deletion syndromes characterized by CPEO, cerebellar ataxia, and dysphagia (Bugiardini et al., 2017). Mutations in *RNASEH1* are still rare and, to date, only 16 patients have been reported (Reyes et al., 2015; Bugiardini et al., 2017; Sachdev et al., 2018; Carreno-Gago et al., 2019). Mutations involve six different residues and are not randomly distributed: four of them are in exon 4, one in exon 5 (in the N-terminal portion of the catalytic domain), and one in exon 3 (in the connecting domain). The fibroblasts from the patient presented here carry two mutations in exon 4: a missense (c.424G > A, p.Val142Ile) and a nonsense mutation (c.469C > T, p.Arg157*). Nonsense mutations are often associated with a decrease in protein level due to nonsense mediated decay (Kurosaki et al., 2019), and this has been reported not only in fibroblasts carrying *RNASEH1* mutations (Reyes et al., 2015) but also in fibroblasts with nonsense mutations in other genes such as *PYCR2* (Zaki et al., 2016), *TIMM50* (Reyes et al., 2018), and *TAOK1* (Dulovic-Mahlow et al., 2019). Similarly, albeit to a lesser degree, two missense mutations in *RNASEH1* (p.Val142Ile and p.Gln86del) have also been described to have an effect on protein stability and therefore to result in a decreased steady-state level of the protein in cultured fibroblasts (Reyes et al., 2015; Akman et al., 2016; Carreno-Gago et al., 2019). In addition, p.Val142Ile mutant RNase H1 activity is only 36-40% of that of wild-type protein based on *in vitro* assays (Reyes et al., 2015; Al-Behadili et al., 2018). With our current knowledge, it is not possible to ascertain whether it is the amino acid substitution itself, the decrease/lack of activity, or a combination of both that is responsible for the observed protein instability.

Notwithstanding the mtDNA depletion and deletion observed in muscle biopsies from patients with mutations in *RNASEH1*, skin fibroblasts derived from the same patients display normal to slightly decreased mtDNA content (Reyes et al., 2015; Akman et al., 2016; Carreno-Gago et al., 2019). Thus, it not surprising that the levels of proteins involved in mtDNA maintenance are not markedly affected either. This could be achieved, on the one hand, by other proteins with similar or complementary functions, such as MGME1, FEN1, and DNA2, helping to maintain the minimum requirements for mtDNA replication, and on the other hand, by changes in the cellular processes like a slow down of cellular growth that could compensate for the slower or less active replication in cells with mutant RNase H1 (Reyes et al., 2015).

Despite the lack of effect on mtDNA, *RNASEH1* mutations have a marked impact on several mitochondrial transcripts in the patient fibroblasts. Significant decreases in mitochondrially encoded complex IV (MT-CO1, MT-CO2, MT-CO3) and complex I (MT-ND1, MT-ND5, MT-ND6) transcripts have been observed in *RNASEH1* patient fibroblasts. However, no such decrease was observed in transcripts from complex III (MT-CYB) and complex V (MT-ATP6) mitochondrial subunits in glucose medium. Although this is the first report of transcript levels in patient fibroblasts, our data are in agreement with recent reports in a *Rnaseh1* liver-specific knockout (KO) mouse model (Lima et al., 2016) and in *Drosophila* S2 cell *rnh1* knockdown (KD) (Gonzalez de Cozar et al., 2019). In the *Rnaseh1* KO mouse, a decline was observed in all mitochondrial transcripts over time from six to 14 weeks of *Rnaseh1* ablation (Lima et al., 2016). In *Drosophila rnh1* KD, transcript levels of Cox3 and ND5 were decreased, while cyt b and ATP8 remained unaltered (Gonzalez de Cozar et al., 2019). The main difference from our patient fibroblasts is that in those cases, mtDNA depletion was also present, making it more difficult to segregate the direct effect of RNAse H1 on transcription from its secondary effect due to partial mtDNA depletion. The non-coding 7S RNA is the only transcript that was increased in the patient fibroblasts in both glucose and galactose medium. This transcript has been described to be involved in the synthesis of the 7S DNA (Gustafsson et al., 2016), and therefore it is not surprising that 7S DNA levels were also increased in the patient fibroblasts, albeit to a much higher level. Much less is known about its role in transcription, despite the fact that early studies suggested that 7S RNA could regulate mitochondrial transcription by preventing the formation of new transcription initiation events (Cantatore et al., 1988). More recently, it has been demonstrated for the first time that RNase H1 is required for the effective removal of 7S RNA, as the *Rnaseh1* KO mouse presents higher levels of 7S RNA, which results in failure to transcribe mtDNA (Lima et al., 2016). In our *RNASEH1* patient fibroblasts, we detected a concomitant increase in 7S RNA and a decrease in seven out of 10 mitochondrial transcripts, supporting the idea that 7S RNA plays a role in their transcription levels. Not only 7S RNA but also other transcripts are able to form R-loops throughout the mitochondrial genome (Brown et al., 2008), and, subsequently, inefficient removal of these structures could block ongoing transcription anywhere along the genome. In spite of this, the mitochondrial degradosome, composed by SUV3 and PNPase, has also been described to be involved in preventing the accumulation of pathological R-loops in mtDNA (Silva et al., 2018), providing a salvage pathway in cells carrying mutations in *RNASEH1*. However, two of the mitochondrial protein transcripts, MT-CYB and MT-ATP6, did not seem to be affected in the patient fibroblasts. This could be explained by a differential transcript half-life, as MT-ATP8/6 transcript is among the longest half-life mitochondrial transcripts in HeLa cells (Nagao et al., 2008). In certain situations, the stabilization of some transcripts could be modified by the up- or downregulation of certain proteins. It has been reported that upon decrease in the steady-state levels of LRPPRC/SLIRP complexes, some transcript levels, including MT-CYB, are less prone to degradation (Chujo et al., 2012). Both LRPPRC and SLIRP were downregulated in patient fibroblasts and therefore could have an effect on MT-CYB transcript stability.

Mitochondrial rRNAs are essential components of the mitochondrial ribosomes, and alterations in their levels often result in mitochondrial translation defects (Boczonadi et al., 2018). The *RNASEH1* patient fibroblasts displayed lower levels of 16S rRNA (MT-RNR2) than controls and, in agreement with these results, lower levels of mitochondrial ribosomal proteins associated with the mt-LSU were observed. This is not the case for 12S rRNA (MT-RNR1) and associated ribosomal proteins, mt-SSU. As discussed above for mitochondrial mRNAs, the steady-state levels of mitochondrial rRNAs can also be modulated by the levels of 7S RNA since this molecule could impede transcription initiation not only at the light but also at the heavy strand promoter (LSP and HSP, respectively). However, this would result in lower levels of both 12S and 16S rRNAs, and we have only detected a decrease in the levels of 16S rRNA. RNase H1, along with P32, has been shown to be involved in the processing of guanosine-cytosine rich mitochondrial ribosomal RNA precursor (12S/16S rRNA precursor) (Wu et al., 2013). Downregulation of RNase H1 increases the levels of the 12S/16S rRNA precursor with one and two species containing 12S and 16S rRNA, respectively (Wu et al., 2013). This suggests that processing of the pre-rRNA by RNase H1 is sequential, originating the mature 12S rRNA in the first step and after further processing, the mature 16S rRNA. A delay in this second processing step could result in the degradation of the partly processed rRNA containing 16S rRNA we observed in the patient fibroblasts. Lower levels of 16S rRNA would result in a decrease of mt-LSU ribosomal proteins, leading to decreased mitochondrial translation. In addition, mitochondrial translation could also be directly modulated by 7S RNA, since this molecule contains a region complementary to the 3’ end of 12S rRNA (Cantatore et al., 1988), and therefore it could alter the structure of the ribosomal subunit, preventing the formation of the full ribosome. A decrease in mitochondrial translation has also been observed in *RNASEH1* patient fibroblasts carrying the p.Val142Ile mutation in homozygosity; however, transcript levels were not analyzed in that case (Akman et al., 2016). The mitochondrial topoisomerase IB (TOP1MT) has also been reported to have a role beyond the resolution of replication and transcription stress, as it has been found to regulate mitochondrial translation through protein–protein interaction with at least one mtSSU ribosomal protein, uS22 (Baechler et al., 2019).

As a result of the alterations in mitochondrial transcription and translation, patient fibroblasts showed OxPhos deficiency with lower oxygen consumption that was not related to mtDNA depletion and slower growth compared to controls. Previous studies also reported lower oxygen consumption in *RNASEH1* patient fibroblasts carrying the p.Val142Ile mutation (Reyes et al., 2015; Akman et al., 2016) and a slower cell growth rate (Reyes et al., 2015; Reyes et al., 2018). However, neither *RNASEH1* patient fibroblasts carrying p.Tyr163His and p.Gln86del mutations (Carreno-Gago et al., 2019) nor *Drosophila rnh1* KD (Gonzalez de Cozar et al., 2019) showed any defect on cell growth. This highlights the fact that *RNASEH1* mutations are rare and subsequently, the number of patients with mutations in this gene is still very low. More comprehensive analyses, including more patient fibroblasts and different mutations, will be needed in order to better establish the role of RNase H1 in mitochondrial transcription and translation and, in particular, the contribution of 7S RNA to these processes.

## Data Availability Statement

The datasets generated for this study are available on request to the corresponding author.

## Ethics Statement

Informed consent for participation in this study was obtained from all investigated subjects in agreement with the Declaration of Helsinki, and the study was approved by the ethical committees of the centers where biological samples were obtained and the Ethical Committee of the Fondazione IRCCS Istituto Neurologico ‘Carlo Besta,’ Milan, Italy.

## Author Contributions

AR conceived the study and designed the experiments, immortalized the skin fibroblasts, performed most of the experiments, interpreted the results, and wrote the manuscript. JR performed some of the experiments. KT critically reviewed the manuscript. MZ provided the funding for the study and critically reviewed the manuscript. All authors read and approved the submitted version of the manuscript.

## Funding

This work was supported by a Core Grant from the MRC (MC_UU_00015/5), ERC Advanced Grant FP7-322424, and NRJ-Institut de France Grant (to MZ). JR was funded by EMBO ASTF 7969 and Polish National Science Center 2014/15/B/NZ5/00434 grants to work under the supervision of AR.

## Conflict of Interest

The authors declare that the research was conducted in the absence of any commercial or financialrelationships that could be construed as a potential conflict of interest.
